# Supplementary material for: Acceptability of a dapivirine levonorgestrel vaginal ring in two Phase 1 trials (MTN-030/IPM 041 and MTN-044/IPM 053/CCN019): Implications for multipurpose prevention technology development
Source: PLoS One. 2025 Jan 14;20(1):e0312957. doi: 10.1371/journal.pone.0312957 (PMC11731730; doi:10.1371/journal.pone.0312957)
Supplement: Attachment — Submitted filename: PONE_Response to Reviewers_25SEPT24.docx [file pone.0312957.s001.docx]

**Response to Reviewers for PONE-D-23-39000**

**Acceptability of a dapivirine levonorgestrel vaginal ring in two Phase 1 trials (MTN-030/IPM 041 and MTN-044/IPM 053/CCN019): implications for multipurpose prevention technology development**

**PLOS ONE**

| **Editor/Reviewer Comment** | **Response** |
| --- | --- |
| **EDITOR’S COMMENTS** | |
| 1. Please ensure that your manuscript meets PLOS ONE's style requirements, including those for file naming. | We have reviewed the manuscript and updated it as relevant per PLOS requirements. |
| 2. Thank you for stating the following in the Competing Interests section:  “Brid Devlin, John Steytler, Kyle Kleinbeck, Bindi Dangi, and Chantél Friend are currently employed by or previously employed by the International Partnership for Microbicides (IPM) that developed the dapivirine levonorgestrel ring. Barbara A. Friedland is employed by Population Council, which acquired IPM's assets in 2022.”  We note that one or more of the authors are employed by a commercial company: International Partnership for Microbicides  a.        Please provide an amended Funding Statement declaring this commercial affiliation, as well as a statement regarding the Role of Funders in your study. If the funding organization did not play a role in the study design, data collection and analysis, decision to publish, or preparation of the manuscript and only provided financial support in the form of authors' salaries and/or research materials, please review your statements relating to the author contributions, and ensure you have specifically and accurately indicated the role(s) that these authors had in your study. You can update author roles in the Author Contributions section of the online submission form.  Please also include the following statement within your amended Funding Statement.  “The funder provided support in the form of salaries for authors [insert relevant initials], but did not have any additional role in the study design, data collection and analysis, decision to publish, or preparation of the manuscript. The specific roles of these authors are articulated in the ‘author contributions’ section.”  If your commercial affiliation did play a role in your study, please state and explain this role within your updated Funding Statement. When submitting your revision, we need you to address these additional requirements. | We have revised and provided an amended funding statement. |
| b. Please also provide an updated Competing Interests Statement declaring this commercial affiliation along with any other relevant declarations relating to employment, consultancy, patents, products in development, or marketed products, etc.  Within your Competing Interests Statement, please confirm that this commercial affiliation does not alter your adherence to all PLOS ONE policies on sharing data and materials by including the following statement: "This does not alter our adherence to PLOS ONE policies on sharing data and materials.” (as detailed online in our guide for authors <http://journals.plos.org/plosone/s/competing-interests>) . If this adherence statement is not accurate and  there are restrictions on sharing of data and/or materials, please state these. Please note that we cannot proceed with consideration of your article until this information has been declared.  Please include both an updated Funding Statement and Competing Interests Statement in your cover letter. We will change the online submission form on your behalf. | We have updated and provided an amended competing interests statement. |
| 3. We note that the grant information you provided in the ‘Funding Information’ and ‘Financial Disclosure’ sections do not match.  When you resubmit, please ensure that you provide the correct grant numbers for the awards you received for your study in the ‘Funding Information’ section. | We have checked and confirmed the grant numbers. |
| 4. In the online submission form, you indicated that [Data can be shared upon request.].  All PLOS journals now require all data underlying the findings described in their manuscript to be freely available to other researchers, either 1. In a public repository, 2. Within the manuscript itself, or 3. Uploaded as supplementary information.  This policy applies to all data except where public deposition would breach compliance with the protocol approved by your research ethics board. If your data cannot be made publicly available for ethical or legal reasons (e.g., public availability would compromise patient privacy), please explain your reasons on resubmission and your exemption request will be escalated for approval. | We have uploaded all of the data to the Population Council’s Dataverse site and will provide a DOI when available. |
| 5. PLOS requires an ORCID iD for the corresponding author in Editorial Manager on papers submitted after December 6th, 2016. Please ensure that you have an ORCID iD and that it is validated in Editorial Manager. To do this, go to ‘Update my Information’ (in the upper left-hand corner of the main menu), and click on the Fetch/Validate link next to the ORCID field. This will take you to the ORCID site and allow you to create a new iD or authenticate a pre-existing iD in Editorial Manager. Please see the following video for instructions on linking an ORCID iD to your Editorial Manager account: [https://www.youtube.com/watch?v=_xcclfuvtxQ](https://nam02.safelinks.protection.outlook.com/?url=https%3A%2F%2Fwww.youtube.com%2Fwatch%3Fv%3D_xcclfuvtxQ&data=05%7C02%7Cbfriedland%40popcouncil.org%7C3d43fba3c6884908a66c08dc5229ab1c%7Cda295b63c8454fa2990b1ab42d54f2ca%7C1%7C0%7C638475586661351588%7CUnknown%7CTWFpbGZsb3d8eyJWIjoiMC4wLjAwMDAiLCJQIjoiV2luMzIiLCJBTiI6Ik1haWwiLCJXVCI6Mn0%3D%7C0%7C%7C%7C&sdata=s1WZW%2FfliRsWcBeoyRHPOjmDvhKyJdqccHSRs%2Fq583U%3D&reserved=0). | The corresponding author’s ORCID ID has now been linked. |
| 6. Please be informed that funding information should not appear in the Acknowledgments section or other areas of your manuscript. We will only publish funding information present in the Funding Statement section of the online submission form. Please remove any funding-related text from the manuscript. | We have removed the funding information from the manuscript. |
| 7. We note that you have included the phrase “data not shown” in your manuscript. Unfortunately, this does not meet our data sharing requirements. PLOS does not permit references to inaccessible data. We require that authors provide all relevant data within the paper, Supporting Information files, or in an acceptable, public repository. Please add a citation to support this phrase or upload the data that corresponds with these findings to a stable repository (such as Figshare or Dryad) and provide and URLs, DOIs, or accession numbers that may be used to access these data. Or, if the data are not a core part of the research being presented in your study, we ask that you remove the phrase that refers to these data. | We have replaced “data not shown” with data on page 32. |
| **REVIEWER #1** |  |
| This manuscript presents acceptability data on the dapivirine levonorgestrel (DPV-LNG) ring from two randomized trials of non-pregnant cisgender women without HIV at the University of Pittsburgh and University of Alabama. Overall, most participants (n=21/23) in a randomized study offering DPV-LNG versus solely DVR for 14 days liked the ring modality they were offered, with no significant difference in acceptability between arm offered DPV-LNG versus DVR-only ring. About half (n=13/25) in the study offering DPV-LNG for 90 days liked the DPV-LNG ring. Those who disliked the ring in MTN-044 reported significantly higher incidence of unanticipated vaginal bleeding, and ring expulsion was reported more frequently by women who disliked the DPV-LNG ring. The overall study is well-conducted, and the authors have identified an important gap in understanding factors impacting combination ring acceptability, adherence, and persistence. The methodology is straightforward, and the results are clearly described. My feedback is described below in detail.  **Major revision:** 1. The described objective of the paper is to explore acceptability of the DPV-LNG ring. However, the acceptability data from MTN-030 presented in Table 3 combines data from both the DVR and DPV-LNG arms, despite much of the data interpretation related to the attributes discussed in Table 3 being separated by arm in the text (lines 309-341). Further, the abstract of the paper asserts that 21/23 women liked the DPV-LNG ring in lines 62-63, when this from my understanding is referencing combined acceptability of both ring methods offered in MTN-030. In addition to modifying this data interpretation in the abstract, would it be possible to make this clearer in Table 3, that MTN-030 is presenting acceptability of both DVR/DPV-LNG, whereas MTN-044 is presenting acceptability of only DPV-LNG options? Alternatively, would the author consider presenting solely the DPV-LNG data from MTN-030 in Table 3? | We thank the reviewer for this comment. We discussed the relative merits of including vs excluding the DVR arm from MTN-030, but felt it was important to keep as it was the comparator in the MTN-030 trial. However, we have made several significant revisions to better articulate the differences in opinions about the DVR vs the DVR-LNG rings within both the text and the tables. The specific revisions we made to address these issues are as follows:   - We added a new table (Table 2) displaying acceptability ratings by randomization group. - We then reorganized the results to present the acceptability by randomization group per study first (the new Table 2), followed by data on adherence and vaginal bleeding by randomization group. - Table 4 is an updated version of the original Table 3, which displays impact of product characteristics, adherence and bleeding on acceptability (like/dislike) of the rings. - We also revised the abstract to make it clear when we are talking about either of the assigned rings vs the DPV-LNG ring. |
| **Minor revision:** 1. Could you please define adherence and persistence as initially presented in line 79? | Thank you for this comment. We define adherence later in the manuscript in the methods section, however, because we did not measure persistence in this trial, we have removed reference to persistence in the introduction. |
| 2. Lines 112-113: Was reporting effective use of condoms considered use of an effective, non-hormonal contraceptive? | Thank you for this comment. Condoms were not considered an effective method in the 14-day MN-030/IPM 041 study. We have clarified this in the manuscript on page 5, line 12. |
| 3. Could you specify which of the measures described in lines 185 through 197 would measure persistence on the DPV-LNG ring, as opposed to adherence, per the above requested definitions of those terms? | As noted above, we removed reference to persistence which was not measured in this study. |
| Reviewer #2: Manuscript is well written and commend the researchers on this great work that is important to inform future development of MPTs. Just minor revision on line 240, there is a punctuation sign missing after the words "due to" as it makes reading of reasons for 4 expulsions confusing when missing. | We thank the reviewer for these comments.  We have edited the text for clarity (lines 243-247) |
| I was struck by the expulsions in MTN044 among women who experienced vaginal bleeding however good enough the authors mention data on expulsions was based off participant report as a limitation. Women experiencing these bleedings could intentionally remove the rings and report this as unintentional expulsion. | We thank the reviewer for this comment and acknowledge that reliance on self-report has the potential to lead to an underreporting of expulsions/non-adherence, whether due to bleeding or other reasons. We mention issues with relying on self-report in the discussion section. |
| Did the participants have ring placement confirmed by attending study staff after first insertion to ensure it is in right place? | Thank you for this question. We have added in the manuscript that if the participant inserted the ring herself, placement was checked by a clinician (lines 122-123; 143-144). |
